# 1,3-Diethyl-1,3-diphenyl­urea

**DOI:** 10.1107/S1600536811008294

**Published:** 2011-03-09

**Authors:** Richard Betz, Thomas Gerber, Henk Schalekamp

**Affiliations:** a Nelson Mandela Metropolitan University, Summerstrand Campus, Department of Chemistry, University Way, Summerstrand, PO Box 77000, Port Elizabeth 6031, South Africa

## Abstract

The mol­ecule of the title compound, C_17_H_20_N_2_O, a symmetrical derivative of urea, shows non-crystallographic *C*_2_ symmetry. Inter­action with the aromatic system of the phenyl substituents as well as amide-type resonance is responsible for the marked planarization of the coordination environments of the N atoms. C—H⋯O contacts give rise to the formation of centrosymmetric dimers in the crystal structure. The closest distance between the centroids of two adjacent rings is 3.8938 (11) Å.

## Related literature

For the crystal structure of a uranium coordination compound with the title compound as a ligand, see: Zhu *et al.* (2008 ▶). For graph-set analysis of hydrogen bonds, see: Etter *et al.* (1990 ▶); Bernstein *et al.* (1995 ▶).
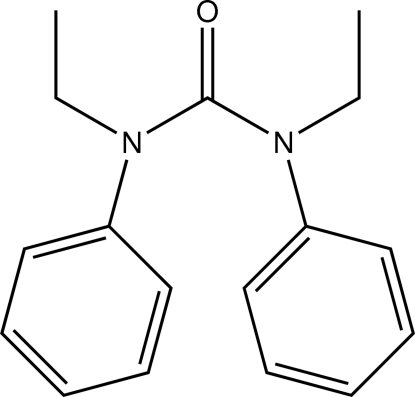


## Experimental

### 

#### Crystal data

C_17_H_20_N_2_O
*M* *_r_* = 268.35
Monoclinic, 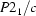

*a* = 9.6990 (5) Å
*b* = 16.7622 (10) Å
*c* = 10.6011 (5) Å
β = 118.854 (4)°
*V* = 1509.52 (14) Å^3^
*Z* = 4
Mo *K*α radiation
μ = 0.07 mm^−1^
*T* = 200 K
0.44 × 0.27 × 0.20 mm

#### Data collection

Bruker APEXII CCD diffractometer
14247 measured reflections
3723 independent reflections
2926 reflections with *I* > 2σ(*I*)
*R*_int_ = 0.030

#### Refinement

*R*[*F* ^2^ > 2σ(*F* ^2^)] = 0.049
*wR*(*F* ^2^) = 0.141
*S* = 1.04
3723 reflections
183 parameters
H-atom parameters constrained
Δρ_max_ = 0.20 e Å^−3^
Δρ_min_ = −0.22 e Å^−3^

### 

Data collection: *APEX2* (Bruker, 2010 ▶); cell refinement: *SAINT* (Bruker, 2010 ▶); data reduction: *SAINT*; program(s) used to solve structure: *SHELXS97* (Sheldrick, 2008 ▶); program(s) used to refine structure: *SHELXL97* (Sheldrick, 2008 ▶); molecular graphics: *ORTEP-3* (Farrugia, 1997 ▶) and *Mercury* (Macrae *et al.*, 2006 ▶); software used to prepare material for publication: *SHELXL97* and *PLATON* (Spek, 2009 ▶).

## Supplementary Material

Crystal structure: contains datablocks I, global. DOI: 10.1107/S1600536811008294/ez2237sup1.cif

Structure factors: contains datablocks I. DOI: 10.1107/S1600536811008294/ez2237Isup2.hkl

Additional supplementary materials:  crystallographic information; 3D view; checkCIF report

## Figures and Tables

**Table 1:** Hydrogen-bond geometry (Å, °)

*D*—H⋯*A*	*D*—H	H⋯*A*	*D*⋯*A*	*D*—H⋯*A*
C13—H13⋯O1^i^	0.95	2.68	3.326 (2)	126

Symmetry code: (i) 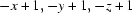.

**Fig. 1.**
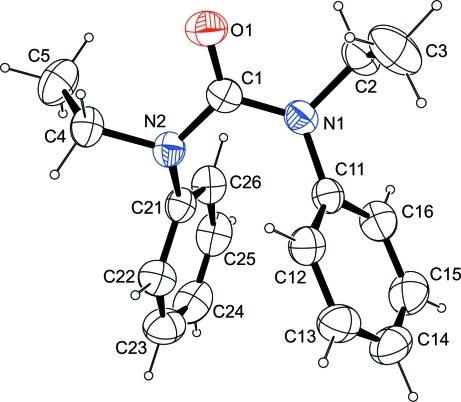
The molecular structure of the title compound, with atom labels and anisotropic displacement ellipsoids (drawn at 50% probability level).

**Fig. 2.**
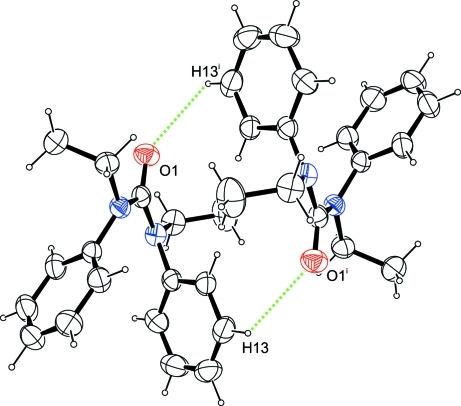
Intermolecular contacts, viewed approximately along [0 1 0]. Symmetry operator: i -x + 1, -y + 1, -z + 1.

**Fig. 3.**
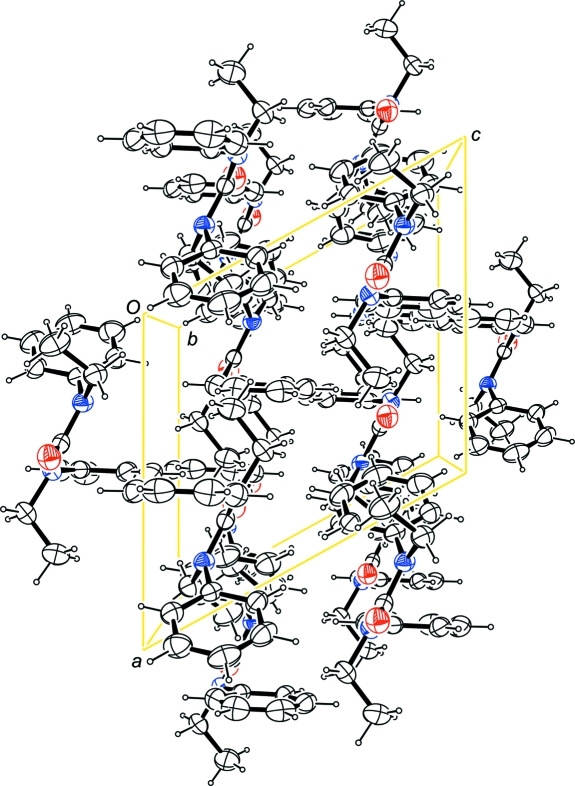
Molecular packing of the title compound, viewed along [0 1 0] (anisotropic displacement ellipsoids drawn at 50% probability level).

## supplementary crystallographic information

### Comment

Chelate ligands are an important class of molecules in coordination chemistry due to the increased thermodynamic stability of coordination compounds obtainable in comparison to those derived from monodentate ligands only. Derivatives of urea are of particular interest in this aspect due to a number of reasons: firstly, urea itself can act as a neutral or – upon deprotonation – anionic ligand. Secondly, apart from exclusive N-donor action, the carbonyl O-atom may serve as donor as well. Thirdly, the possible derivatization of the N-atoms allows for the fine-tuning of the nucleophilicity of these atoms, such that a vast series of different symmetric, as well as asymmetric, derivatives is at hand. A crystal structure of a uranium coordination compound utilising the title compound as ligand has been published (Zhu *et al.*, 2008). At the beginning of a bigger study to elucidate the rules guiding the formation of urea-derivative-supported coordination compounds we determined the structure of the title compound to allow for comparisons with the ligand in such compounds.

In the molecule (Fig. 1), the coordination environment around both nitrogen atoms is almost planar due to the interaction of the free electron pair not only in terms of amide-type resonance but also with the phenyl-moiety. The N-atoms are displaced by only 0.183 (1) Å and -0.180 (1) Å from the planes defined by the atoms bonded to them.

The least-squares planes defined by the carbon atoms of the phenyl rings enclose an angle of 40.31 (4) °.

In the crystal structure, C–H···O contacts are present whose range falls sightly below the sum of van-der-Waals radii of the atoms involved. They involve one of the phenyl hydrogen atoms which is *meta* to the nitrogen atom and connect the molecules into centrosymmetric dimers (Fig. 2). In terms of graph-set analysis, the descriptor for these contacts on the unitary level is *R*^2^_2_(14) (Etter *et al.*, 1990; Bernstein *et al.*, 1995). The closest distance between two centers of gravity was measured at 3.8938 (11) Å.

The packing of the title compound is shown in Fig. 3.

### Experimental

The compound was obtained commercially (Aldrich). Crystals suitable for the X-ray diffraction study were taken directly from the provided compound.

### Refinement

Carbon-bound H-atoms were placed in calculated positions (C—H 0.99 Å for methylene groups, C—H 0.95 Å for aromatic C-atoms) and were included in the refinement in the riding model approximation, with *U*(H) set to 1.2*U*_eq_(C). The H atoms of the methyl groups were allowed to rotate with a fixed angle around the C—C bond to best fit the experimental electron density (HFIX 137 in the *SHELX* program suite (Sheldrick, 2008)), with *U*(H) set to 1.5*U*_eq_(C).

### Crystal data

**Table:**

C_17_H_20_N_2_O	*F*(000) = 576
*M**_r_* = 268.35	*D*_x_ = 1.181 Mg m^−^^3^
Monoclinic, *P*2_1_/*c*	Mo *K*α radiation, λ = 0.71073 Å
Hall symbol: -P 2ybc	Cell parameters from 6636 reflections
*a* = 9.6990 (5) Å	θ = 2.5–28.2°
*b* = 16.7622 (10) Å	µ = 0.07 mm^−^^1^
*c* = 10.6011 (5) Å	*T* = 200 K
β = 118.854 (4)°	Platelet, colourless
*V* = 1509.52 (14) Å^3^	0.44 × 0.27 × 0.20 mm
*Z* = 4	

### Data collection

**Table:**

Bruker APEXII CCD diffractometer	2926 reflections with *I* > 2σ(*I*)
Radiation source: fine-focus sealed tube	*R*_int_ = 0.030
graphite	θ_max_ = 28.3°, θ_min_ = 2.4°
φ and ω scans	*h* = −12→8
14247 measured reflections	*k* = −22→22
3723 independent reflections	*l* = −13→14

### Refinement

**Table:**

Refinement on *F*^2^	Primary atom site location: structure-invariant direct methods
Least-squares matrix: full	Secondary atom site location: difference Fourier map
*R*[*F*^2^ > 2σ(*F*^2^)] = 0.049	Hydrogen site location: inferred from neighbouring sites
*wR*(*F*^2^) = 0.141	H-atom parameters constrained
*S* = 1.04	*w* = 1/[σ^2^(*F*_o_^2^) + (0.0689*P*)^2^ + 0.3719*P*] where *P* = (*F*_o_^2^ + 2*F*_c_^2^)/3
3723 reflections	(Δ/σ)_max_ < 0.001
183 parameters	Δρ_max_ = 0.20 e Å^−^^3^
0 restraints	Δρ_min_ = −0.22 e Å^−^^3^

### Fractional atomic coordinates and isotropic or equivalent isotropic displacement parameters (Å^2^)

**Table:**

	*x*	*y*	*z*	*U*_iso_*/*U*_eq_	
O1	0.27619 (13)	0.58513 (6)	0.22318 (11)	0.0467 (3)	
N1	0.31375 (13)	0.45259 (7)	0.20565 (11)	0.0369 (3)	
N2	0.16310 (13)	0.49836 (7)	0.31074 (11)	0.0351 (3)	
C1	0.25214 (15)	0.51597 (8)	0.24532 (13)	0.0345 (3)	
C2	0.39920 (18)	0.47225 (10)	0.12691 (15)	0.0463 (4)	
H2A	0.3518	0.5205	0.0678	0.056*	
H2B	0.3868	0.4278	0.0607	0.056*	
C3	0.5722 (2)	0.48692 (15)	0.2249 (2)	0.0713 (6)	
H3A	0.5854	0.5310	0.2905	0.107*	
H3B	0.6227	0.5006	0.1671	0.107*	
H3C	0.6208	0.4386	0.2810	0.107*	
C4	0.10811 (17)	0.56703 (9)	0.36121 (16)	0.0421 (3)	
H4A	0.1906	0.6088	0.3975	0.050*	
H4B	0.0916	0.5499	0.4423	0.050*	
C5	−0.04350 (19)	0.60209 (11)	0.2438 (2)	0.0592 (5)	
H5A	−0.0279	0.6192	0.1632	0.089*	
H5B	−0.0740	0.6481	0.2819	0.089*	
H5C	−0.1268	0.5617	0.2102	0.089*	
C11	0.35486 (15)	0.37776 (8)	0.27978 (14)	0.0352 (3)	
C12	0.43976 (16)	0.37458 (9)	0.42910 (15)	0.0395 (3)	
H12	0.4658	0.4225	0.4838	0.047*	
C13	0.48642 (18)	0.30161 (10)	0.49829 (18)	0.0496 (4)	
H13	0.5423	0.2994	0.6005	0.060*	
C14	0.4519 (2)	0.23230 (10)	0.4191 (2)	0.0580 (4)	
H14	0.4854	0.1823	0.4666	0.070*	
C15	0.3686 (2)	0.23543 (10)	0.2708 (2)	0.0579 (4)	
H15	0.3451	0.1875	0.2165	0.069*	
C16	0.31902 (18)	0.30777 (9)	0.20057 (17)	0.0469 (4)	
H16	0.2606	0.3095	0.0984	0.056*	
C21	0.07073 (15)	0.42715 (8)	0.28284 (14)	0.0350 (3)	
C22	0.07456 (17)	0.38606 (9)	0.39755 (16)	0.0437 (3)	
H22	0.1423	0.4033	0.4934	0.052*	
C23	−0.0204 (2)	0.31984 (10)	0.3726 (2)	0.0557 (4)	
H23	−0.0178	0.2916	0.4514	0.067*	
C24	−0.1193 (2)	0.29477 (10)	0.2329 (2)	0.0588 (5)	
H24	−0.1843	0.2492	0.2159	0.071*	
C25	−0.12340 (18)	0.33586 (10)	0.11852 (18)	0.0531 (4)	
H25	−0.1911	0.3185	0.0228	0.064*	
C26	−0.02929 (16)	0.40234 (9)	0.14263 (15)	0.0436 (3)	
H26	−0.0330	0.4309	0.0636	0.052*	

### Atomic displacement parameters (Å^2^)

**Table:**

	*U*^11^	*U*^22^	*U*^33^	*U*^12^	*U*^13^	*U*^23^
O1	0.0549 (6)	0.0398 (6)	0.0458 (6)	−0.0044 (5)	0.0245 (5)	0.0027 (4)
N1	0.0368 (6)	0.0425 (6)	0.0333 (5)	0.0007 (5)	0.0185 (5)	0.0019 (4)
N2	0.0330 (6)	0.0350 (5)	0.0370 (5)	−0.0016 (4)	0.0166 (5)	−0.0042 (4)
C1	0.0307 (6)	0.0398 (7)	0.0275 (5)	−0.0015 (5)	0.0095 (5)	−0.0001 (5)
C2	0.0504 (8)	0.0574 (9)	0.0387 (7)	0.0007 (7)	0.0274 (6)	0.0046 (6)
C3	0.0507 (10)	0.1048 (17)	0.0672 (11)	−0.0138 (10)	0.0355 (9)	0.0088 (11)
C4	0.0420 (7)	0.0397 (7)	0.0457 (7)	0.0000 (6)	0.0220 (6)	−0.0079 (6)
C5	0.0423 (9)	0.0490 (9)	0.0782 (12)	0.0073 (7)	0.0227 (8)	−0.0042 (8)
C11	0.0296 (6)	0.0394 (7)	0.0389 (7)	−0.0010 (5)	0.0182 (5)	−0.0010 (5)
C12	0.0321 (6)	0.0434 (7)	0.0403 (7)	0.0003 (5)	0.0153 (5)	−0.0004 (6)
C13	0.0378 (8)	0.0569 (9)	0.0506 (8)	0.0054 (6)	0.0187 (6)	0.0144 (7)
C14	0.0532 (9)	0.0419 (8)	0.0867 (13)	0.0050 (7)	0.0399 (9)	0.0157 (8)
C15	0.0627 (11)	0.0393 (8)	0.0792 (12)	−0.0080 (7)	0.0403 (10)	−0.0095 (8)
C16	0.0466 (8)	0.0465 (8)	0.0491 (8)	−0.0080 (6)	0.0244 (7)	−0.0091 (6)
C21	0.0280 (6)	0.0362 (6)	0.0396 (7)	0.0003 (5)	0.0152 (5)	−0.0028 (5)
C22	0.0393 (7)	0.0471 (8)	0.0429 (7)	−0.0026 (6)	0.0184 (6)	0.0012 (6)
C23	0.0511 (9)	0.0507 (9)	0.0676 (10)	−0.0046 (7)	0.0304 (8)	0.0086 (8)
C24	0.0416 (8)	0.0441 (9)	0.0864 (13)	−0.0099 (7)	0.0274 (9)	−0.0094 (8)
C25	0.0372 (8)	0.0554 (9)	0.0562 (9)	−0.0066 (7)	0.0141 (7)	−0.0176 (8)
C26	0.0341 (7)	0.0509 (8)	0.0393 (7)	−0.0012 (6)	0.0125 (6)	−0.0056 (6)

### Geometric parameters (Å, °)

**Table:**

O1—C1	1.2273 (16)	C12—C13	1.385 (2)
N1—C1	1.3803 (17)	C12—H12	0.9500
N1—C11	1.4310 (17)	C13—C14	1.377 (3)
N1—C2	1.4697 (17)	C13—H13	0.9500
N2—C1	1.3755 (18)	C14—C15	1.379 (3)
N2—C21	1.4349 (17)	C14—H14	0.9500
N2—C4	1.4734 (17)	C15—C16	1.382 (2)
C2—C3	1.507 (2)	C15—H15	0.9500
C2—H2A	0.9900	C16—H16	0.9500
C2—H2B	0.9900	C21—C22	1.382 (2)
C3—H3A	0.9800	C21—C26	1.3899 (19)
C3—H3B	0.9800	C22—C23	1.384 (2)
C3—H3C	0.9800	C22—H22	0.9500
C4—C5	1.514 (2)	C23—C24	1.385 (3)
C4—H4A	0.9900	C23—H23	0.9500
C4—H4B	0.9900	C24—C25	1.378 (3)
C5—H5A	0.9800	C24—H24	0.9500
C5—H5B	0.9800	C25—C26	1.384 (2)
C5—H5C	0.9800	C25—H25	0.9500
C11—C16	1.3859 (19)	C26—H26	0.9500
C11—C12	1.3887 (19)		
			
C1—N1—C11	123.66 (11)	C12—C11—N1	120.96 (12)
C1—N1—C2	116.50 (12)	C13—C12—C11	120.00 (14)
C11—N1—C2	115.12 (11)	C13—C12—H12	120.0
C1—N2—C21	123.78 (11)	C11—C12—H12	120.0
C1—N2—C4	116.15 (11)	C14—C13—C12	120.06 (15)
C21—N2—C4	115.16 (11)	C14—C13—H13	120.0
O1—C1—N2	121.51 (12)	C12—C13—H13	120.0
O1—C1—N1	121.23 (12)	C13—C14—C15	119.97 (15)
N2—C1—N1	117.25 (11)	C13—C14—H14	120.0
N1—C2—C3	112.95 (12)	C15—C14—H14	120.0
N1—C2—H2A	109.0	C14—C15—C16	120.51 (15)
C3—C2—H2A	109.0	C14—C15—H15	119.7
N1—C2—H2B	109.0	C16—C15—H15	119.7
C3—C2—H2B	109.0	C15—C16—C11	119.73 (15)
H2A—C2—H2B	107.8	C15—C16—H16	120.1
C2—C3—H3A	109.5	C11—C16—H16	120.1
C2—C3—H3B	109.5	C22—C21—C26	120.02 (13)
H3A—C3—H3B	109.5	C22—C21—N2	118.95 (12)
C2—C3—H3C	109.5	C26—C21—N2	120.90 (13)
H3A—C3—H3C	109.5	C21—C22—C23	119.91 (14)
H3B—C3—H3C	109.5	C21—C22—H22	120.0
N2—C4—C5	112.51 (12)	C23—C22—H22	120.0
N2—C4—H4A	109.1	C22—C23—C24	120.08 (16)
C5—C4—H4A	109.1	C22—C23—H23	120.0
N2—C4—H4B	109.1	C24—C23—H23	120.0
C5—C4—H4B	109.1	C25—C24—C23	120.01 (15)
H4A—C4—H4B	107.8	C25—C24—H24	120.0
C4—C5—H5A	109.5	C23—C24—H24	120.0
C4—C5—H5B	109.5	C24—C25—C26	120.23 (15)
H5A—C5—H5B	109.5	C24—C25—H25	119.9
C4—C5—H5C	109.5	C26—C25—H25	119.9
H5A—C5—H5C	109.5	C25—C26—C21	119.75 (15)
H5B—C5—H5C	109.5	C25—C26—H26	120.1
C16—C11—C12	119.71 (13)	C21—C26—H26	120.1
C16—C11—N1	119.17 (12)		
			
C21—N2—C1—O1	149.90 (12)	C11—C12—C13—C14	1.5 (2)
C4—N2—C1—O1	−4.06 (18)	C12—C13—C14—C15	−1.0 (2)
C21—N2—C1—N1	−30.20 (17)	C13—C14—C15—C16	−0.1 (3)
C4—N2—C1—N1	175.84 (11)	C14—C15—C16—C11	0.7 (2)
C11—N1—C1—O1	150.16 (13)	C12—C11—C16—C15	−0.3 (2)
C2—N1—C1—O1	−4.23 (18)	N1—C11—C16—C15	175.11 (14)
C11—N1—C1—N2	−29.74 (17)	C1—N2—C21—C22	136.14 (14)
C2—N1—C1—N2	175.87 (11)	C4—N2—C21—C22	−69.67 (16)
C1—N1—C2—C3	89.43 (18)	C1—N2—C21—C26	−48.07 (18)
C11—N1—C2—C3	−67.16 (19)	C4—N2—C21—C26	106.12 (15)
C1—N2—C4—C5	85.17 (16)	C26—C21—C22—C23	0.5 (2)
C21—N2—C4—C5	−71.05 (16)	N2—C21—C22—C23	176.32 (13)
C1—N1—C11—C16	138.84 (14)	C21—C22—C23—C24	0.0 (2)
C2—N1—C11—C16	−66.45 (16)	C22—C23—C24—C25	−0.1 (3)
C1—N1—C11—C12	−45.86 (18)	C23—C24—C25—C26	−0.2 (3)
C2—N1—C11—C12	108.86 (15)	C24—C25—C26—C21	0.6 (2)
C16—C11—C12—C13	−0.9 (2)	C22—C21—C26—C25	−0.8 (2)
N1—C11—C12—C13	−176.13 (13)	N2—C21—C26—C25	−176.53 (13)

### Hydrogen-bond geometry (Å, °)

**Table:**

*D*—H···*A*	*D*—H	H···*A*	*D*···*A*	*D*—H···*A*
C13—H13···O1^i^	0.95	2.68	3.326 (2)	126

Symmetry codes: (i) −*x*+1, −*y*+1, −*z*+1.
